# Rooftop Breeding Does Not Consistently Explain Variation in Yolk Testosterone in Herring Gulls

**DOI:** 10.1002/ece3.74269

**Published:** 2026-09-06

**Authors:** Jolien Van Malderen, Luc Lens, Frederick Verbruggen, Eric W. M. Stienen, Wendt Müller

**Affiliations:** ^1^ Centre for Research on Ecology, Cognition and Behaviour of Birds Ghent University Ghent Belgium; ^2^ Behavioural Ecology and Ecophysiology Research Group University of Antwerp Antwerp Belgium; ^3^ Research Institute for Nature and Forest (INBO) Brussels Belgium

**Keywords:** androgen allocation, egg hormones, *Larus argentatus*, maternal effects, urbanisation, yolk testosterone

## Abstract

In oviparous species, females shape offspring phenotype during early development by varying the resources they deposit in the egg prior to laying. Yolk androgen levels, for example, vary within and among females and their deposition is influenced by maternal state, as well as environmental conditions, including the social environment. By providing artificial breeding sites, such as rooftops, urbanisation has created novel breeding contexts for Herring gulls, which, however, differ from a traditional ground‐breeding context in several ecological characteristics that may influence yolk androgen deposition. Here, we compared yolk testosterone concentrations in the last‐laid egg of rooftop‐ and ground‐breeding Herring gulls across two breeding seasons and tested whether variation was associated with social environment (approximated by nest density) or maternal quality differences (reflected by egg volume, laying date of the last egg and diet composition). Rooftop‐breeding pairs showed higher yolk testosterone concentrations than ground‐breeding pairs in 2023, while no difference was observed in 2024. Ground‐breeding pairs also exhibited significant interannual variation, whereas rooftop pairs did not. Neither nest density nor any proxy of maternal quality explained variation in yolk testosterone levels. These results indicate that yolk testosterone allocation in Herring gulls is variable but not consistently explained by breeding context, social environment or maternal state.

## Graphical abstract

Rooftop‐ and ground‐breeding represent distinct ecological contexts that may influence maternal hormone allocation in Herring gulls. Yolk testosterone concentrations differed between rooftop‐ and ground‐breeding pairs in one of 2 years but were not consistently associated with breeding context, nest density or proxies of maternal quality, suggesting that variation in maternal hormone allocation is not systematically explained by these factors.

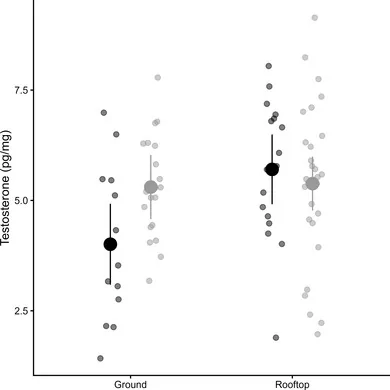

## 1 Introduction

In oviparous species, embryonic development occurs outside the maternal body within the confines of a sealed egg. All resources required for the embryo, such as lipids, proteins, vitamins and minerals, must therefore be deposited prior to laying (Romanoff and Romanoff 1949). Variation in yolk composition can thus have important consequences for early development. In particular, yolk testosterone has received considerable attention due to its potent, non‐genetic effects during this stage, with lasting post‐hatch phenotypic consequences (Schwabl 1993; Gil 2003; Groothuis et al. 2005). Specifically, experimentally elevated yolk testosterone levels have been shown to result in shorter incubation periods, faster post‐hatching growth, increased begging behaviour, enhanced competitive ability and increased chick survival (Schwabl 1996; Eising and Groothuis 2003; Navara et al. 2006a).

However, substantial variation exists both within and among clutches in yolk testosterone concentrations. Within clutches, variation is often linked to laying order: in species with asynchronous hatching, later‐laid eggs frequently contain higher testosterone concentrations (Schwabl 1993; Groothuis and Schwabl 2002; Tanvez et al. 2008). In addition, considerable variation occurs among females, reflecting both intrinsic and extrinsic factors (Müller et al. 2002; Mazuc et al. 2003; Pilz et al. 2003; Verboven et al. 2003; Pilz and Smith 2004; Navara et al. 2006b; Guibert et al. 2012; Widowski et al. 2022). For example, females in poor body condition or paired with less attractive males have been shown to deposit higher testosterone concentrations (Verboven et al. 2003; Navara et al. 2006b). Ecological factors associated with increased social stimulation, such as high breeding density, are often positively related to yolk testosterone levels (James and Bentz 2024). Together, these patterns indicate that yolk testosterone deposition is flexible and responsive to both maternal state and environmental conditions.

Because yolk testosterone has pronounced effects on offspring phenotype and performance, such variation in its deposition is likely to have important fitness consequences. Two non‐mutually exclusive explanations have been proposed to account for this variation. First, elevated testosterone deposition may serve a compensatory function. For example, the elevated testosterone concentrations in eggs laid later in the laying sequence, or in eggs of females in poor condition or paired with less attractive males, may mitigate developmental disadvantages experienced by the offspring (Schwabl 1993; Eising et al. 2001). Alternatively, or additionally, variation in yolk testosterone may reflect adaptive responses to predictable environmental conditions. For instance, increased testosterone deposition under high levels of social interaction may prepare offspring for more competitive environments (James and Bentz 2024).

If yolk testosterone reflects adaptive responses to environmental conditions, it may also be sensitive to anthropogenic environmental change, including human‐altered habitats. Urbanisation, one of the most pervasive and rapid forms of anthropogenic environmental change (Seress and Liker 2015), creates novel breeding opportunities for many species, which, however, differ in ecological conditions from the natural breeding sites (Marzluff 2001; Reynolds et al. 2019). Examining whether females adjust maternal hormone allocation in response to these altered ecological conditions can therefore provide insight into their capacity to adapt offspring phenotype to match these novel contexts.

One novel breeding context associated with urban environments is nesting on man‐made structures. In Herring gulls ( *Larus argentatus* ), for example, individuals frequently breed on rooftops within urban environments (Stienen et al. 2002; Rock 2005), where ecological conditions differ from those experienced in traditional ground colonies. For example, breeding density is typically lower on rooftops than in ground colonies (Monaghan 1979; Kroc 2018; Pais De Faria et al. 2023), which likely translates into differences in levels of aggression, competition and social interaction among breeding females. Given that social stimuli are among the strongest ecological predictors of yolk testosterone deposition (James and Bentz 2024), this may in turn influence maternal testosterone allocation (Mazuc et al. 2003). In addition, rooftop and ground colonies differ in several other ecological characteristics, including predation pressure, human disturbance and microclimate. These ecological differences may alter the environmental conditions and cues experienced by breeding females, potentially affecting their physiological state and, consequently, maternal hormone allocation. For instance, rooftops may disproportionately attract less competitive females or impose dietary constraints through increased access to anthropogenic food sources, which are highly predictable in space and time (Oro et al. 2013) but may differ in nutritional quality from natural resources (Pierotti and Annett 1991; Bernat‐Ponce et al. 2023). Such differences in maternal condition or resource quality could further shape hormone allocation patterns.

Despite these multiple ecological differences between rooftop‐ and ground‐breeding habitats, it remains unclear whether maternal testosterone allocation differs between these breeding contexts and, if so, which ecological factors contribute to any observed variation. Understanding such variation may help to clarify how maternal hormone allocation responds to environmental variation in human‐altered habitats.

In this study, we compared maternal yolk testosterone allocation between ground‐ and rooftop‐breeding Herring gulls and explored the ecological drivers that may underlie any observed differences. We specifically evaluated the potential roles of local social environment and maternal characteristics. Because yolk testosterone is generally positively associated with competitive interactions, although this relationship may be weaker in colonial species (Bentz et al. 2016), we predicted that eggs laid by rooftop‐breeding females would contain lower testosterone concentrations than those laid in ground colonies, where breeding densities are higher. In addition, because maternal state may also influence yolk testosterone allocation, we tested whether females in poorer condition deposited higher testosterone concentrations, potentially as a compensatory response to reduced offspring developmental prospects. For this purpose, we used egg size, laying date and reliance on high‐quality marine food sources (Pierotti and Annett 1991), estimated using stable isotope analysis, as complementary proxies for maternal quality, each reflecting different aspects of maternal state. Egg size is often positively related to female condition, as females in better condition generally produce larger eggs (Murphy 1986; Reid and Boersma 1990). Similarly, earlier laying is frequently associated with higher‐quality females, reflecting their ability to acquire sufficient resources and secure high‐quality breeding sites, enabling earlier breeding (Moreno et al. 1998; Descamps et al. 2011; Bosman et al. 2013). Finally, because marine prey are generally considered nutritionally superior for Herring gulls (Pierotti and Annett 1991), greater reliance on marine resources may indicate access to higher‐quality food and consequently better maternal condition.

## 2 Materials and Methods

### 2.1 General Field Methods

We compared Herring gull breeding pairs across contrasting breeding contexts. Fieldwork was conducted during the breeding seasons of 2023 and 2024 (late April–July). Ground‐nesting Herring gulls were monitored in a semi‐natural mixed‐species breeding colony within the outer port of Zeebrugge, Belgium, where Herring gulls breed among Lesser black‐backed gulls ( *Larus fuscus* ). The colony represents a ground‐breeding context within a human‐modified landscape and is divided into three adjacent fenced sections separated by a road, which were treated as a single breeding colony throughout this study. This colony comprised 347 breeding pairs in 2023 and 335 in 2024, including 29 and 27 Herring gull pairs respectively. Rooftop‐breeding gulls were monitored at multiple sites within the city of Ostend, Belgium. Buildings were selected prior to the breeding season based on practical and safety considerations: only flat roofs were included to ensure safe access, and sites were restricted to public or commercial buildings (e.g., schools, companies and government buildings) to allow repeated access without reliance on private residents. The selected buildings represent common types of urban rooftop habitats used by breeding gulls in the study area. An overview of sampling locations and collected samples is provided in Table A1.

Nests were checked three times per week (Monday, Wednesday, Friday) to determine laying dates. As Herring gulls typically lay three‐egg clutches at approximately two‐day intervals (Drent 1970), this monitoring frequency allowed reliable identification of laying order in most cases. Eggs were marked upon discovery with permanent marker to indicate laying order: ‘1’, ‘2’ or ‘3’ for known laying order, ‘A’, ‘B’ or ‘C’ when laying order was unknown. In clutches with unknown laying order, the last‐laid egg was identified by its more pronounced streaked spotting pattern (Chamberlin 1977). To account for potential resampling across years, breeding pairs were assigned unique Pair IDs. Resampled pairs were identified via colour‐rings, or, when not ringed, by repeated occupancy of the same nest location in both years. Because Herring gulls exhibit high pair and nest site fidelity (Bongiorno 1970; Fitch and Shugart 1983), we consider repeated occupancy of the same nest location and repeated observation of one colour‐ringed pair member reasonable criteria for identifying repeated breeding pairs across years. Out of 68 unique pairs sampled in total, 11 breeding pairs were sampled in both years (seven ground‐breeding and four rooftop‐breeding pairs).

### 2.2 Egg Collection and Processing

Eggs were measured and collected to obtain estimates of maternal condition and yolk samples for testosterone analysis. Upon discovery of a completed clutch (i.e., after the third egg had been laid), all eggs were measured (±0.1 mm) using callipers. Egg volume was calculated as: 0.476 × L × W^2^/1000 (Harris 1964), where L is egg length and W is egg width. The last‐laid egg was collected immediately upon discovery of the completed clutch, under licence from the Agency for Nature and Forests (Agentschap voor Natuur en Bos, ANB). Given the nest visit frequency (Monday, Wednesday, Friday), collection typically occurred within 1–2 days of laying. Because yolk testosterone increases with laying order in gulls (Eising et al. 2001), we standardised egg collection, analysing only eggs of the same laying position to obtain a reliable estimate of maternal yolk deposition. The last‐laid egg was selected because it generally has the lowest probability of producing a fledgling within the clutch (Parsons 1970), thereby minimising impacts on reproductive success. Moreover, collecting the last‐laid egg minimises disruption to the breeding attempt, as removal of this egg leaves the remaining clutch intact for continued incubation. Eggs were stored frozen until further processing. During sample preparation, eggs were partially thawed to remove the eggshell and separate yolk and albumen. Eggs showing visible embryonic development were excluded from analyses, as embryonic development may alter yolk steroid concentrations. In total, 14 ground and 17 rooftop yolk samples from 2023, and 20 ground and 28 rooftop yolk samples from 2024 were analysed. To obtain representative samples, each yolk was halved while still frozen, ensuring both inner and outer yolk layers were present in each sample, as steroid deposition varies within the yolk (Lipar et al. 1999). One half was used for testosterone analysis, while the other half was retained for additional analyses not presented here.

### 2.3 Nest Density

Local nest density was quantified as a proxy for the social environment experienced by the females. Coordinates of ground nests were recorded using an RTK GNSS receiver (Carlson BRx7). Rooftop nest locations were determined using an online map and extracted as geographic coordinates. Inter‐nest distances were calculated from latitude and longitude coordinates. Local nest density was quantified for each individual nest as the number of neighbouring nests (either Herring gull or Lesser black‐backed gull) within a 5 m radius. This metric was selected because a 5 m radius corresponds to the scale at which most agonistic interactions occur in breeding gulls (Hutson 1977), and therefore captures the number of individuals with which direct social interactions are most likely. We included both Herring gull and Lesser black‐backed gull neighbours because they breed sympatrically in our study sites and engage in competitive and aggressive interactions, such that heterospecific neighbours contribute to the local social environment experienced by breeding Herring gulls (Garthe et al. 1999).

### 2.4 Testosterone Assays

Yolk testosterone concentrations were quantified using enzyme immunoassays following extraction of steroid hormones from yolk samples. Yolk hormone extraction followed Gil et al. (2004) with minor modifications. Each yolk sample was weighed to the nearest 0.001 g and diluted (1:2 w/v) in double distilled water, and half of the homogenised sample was used for steroid extraction. Steroids were extracted by adding 3 mL of a 30:70 petroleum ether: diethyl ether mixture to the diluted yolk sample. Samples were vortexed for 10 min and centrifuged for 10 min at 4°C (1500 rpm). The ether phase was recovered after snap‐freezing the tube in an ethanol bath with dry ice. The extraction procedure was repeated a second time, and the ether phases were combined. Combined extracts were evaporated to dryness at 50°C under a stream of nitrogen. To remove protein residues, the dried extract was resuspended in 0.5 mL of 90% ethanol, vigorously mixed and stored overnight at −20°C. The following day, samples were centrifuged (10 min at 4°C, 1500 rpm) and the supernatant was transferred to a clean tube. Extracts were again evaporated to dryness and resuspended in 0.5 mL steroid‐free serum (DRG Labs, Germany). After vortexing for 10 min, samples were transferred to Eppendorf tubes and stored at −20°C until hormone quantification.

Testosterone concentrations were determined using an enzyme immunoassay according to the manufacturer's protocol (Testosterone ELISA; ref. EIA‐1559; DRG, Germany). All samples were analysed in duplicate. Inter‐ and intra‐assay variability were assessed using coefficients of variation (CV), which were 7.90% and 3.53% respectively. Absorbance was measured at 450 nm using a Synergy HT Multi‐Mode Microplate Reader (Biotek, USA) according to EIA procedure. Final testosterone concentrations were adjusted for yolk mass to obtain values expressed per egg (pg/mg).

### 2.5 Stable Isotope Analysis

Maternal diet during egg formation was estimated using stable isotope analysis of chick down feathers. Nests were monitored throughout incubation to determine egg loss and hatch dates. Upon hatching, down feathers were collected from chicks for stable isotope analysis. Because hatchling down is synthesised entirely from nutrients deposited in the egg by the mother, its carbon (δ^13^C) and nitrogen (δ^15^N) isotope composition reflects maternal diet during egg formation (Klaassen et al. 2004). Carbon and nitrogen isotope ratios are highly repeatable among siblings, allowing inference of maternal diet for the collected egg (Allen et al. 2025). Feathers were rinsed in a 2:1 chloroform: methanol solution to remove surface oils, air‐dried under a fume hood and placed into tin capsules for analysis. Isotope ratios were obtained using a Micro Vario elemental analyser (Elementar, Germany), coupled with a PrecisION isotopic ratio mass spectrometer (Elementar, Germany) at the Laboratoire d'Ecologie Trophique et Isotopique (LETIS), University of Liège (Liège, Belgium). Data were expressed in the δ notation (‰) (Equation 1) relative to international standards, namely the Vienna Pee Dee Belemnite (PDB) for carbon and N_2_ in atmospheric air for nitrogen:

(1) $$\delta X_{\text{sample}}=\left[\frac{{\left(X/x\right)}_{\text{sample}}}{{\left(X/x\right)}_{\text{standard}}}-1\right]\times 1000$$

where *X* is the heavy isotope (^13^C or ^15^N), *x* is the lighter isotope (^12^C or ^14^N) and (*X*/*x*)_sample_ and (*X*/*x*)_standard_ are the ratios of both stable isotopes in the sample and the standard, respectively. Certified reference materials from the International Atomic Energy Agency (IAEA), IAEA—600 (caffeine; δ^15^N = 1.04‰ ± 0.05‰; δ^13^C = −27.7‰ ± 0.05‰; mean ± SD) and IAEA C‐6 (sucrose; δ^13^C = −10.8‰ ± 0.5‰) were included in the batch. Laboratory glycine (Sigma‐Aldrich; δ^13^C = −47.3‰ ± 0.3‰; δ^15^N = 2.4‰ ± 0.3‰) was used as quality control every 15 samples. Replicated measurements of mussel sample (δ^15^N = 3.73‰; δ^13^C = −21.59‰), spread every 15 samples provided a standard deviation of ±0.07‰ and ±0.09‰ for δ^13^C and δ^15^N, respectively.

**FIGURE A1:**
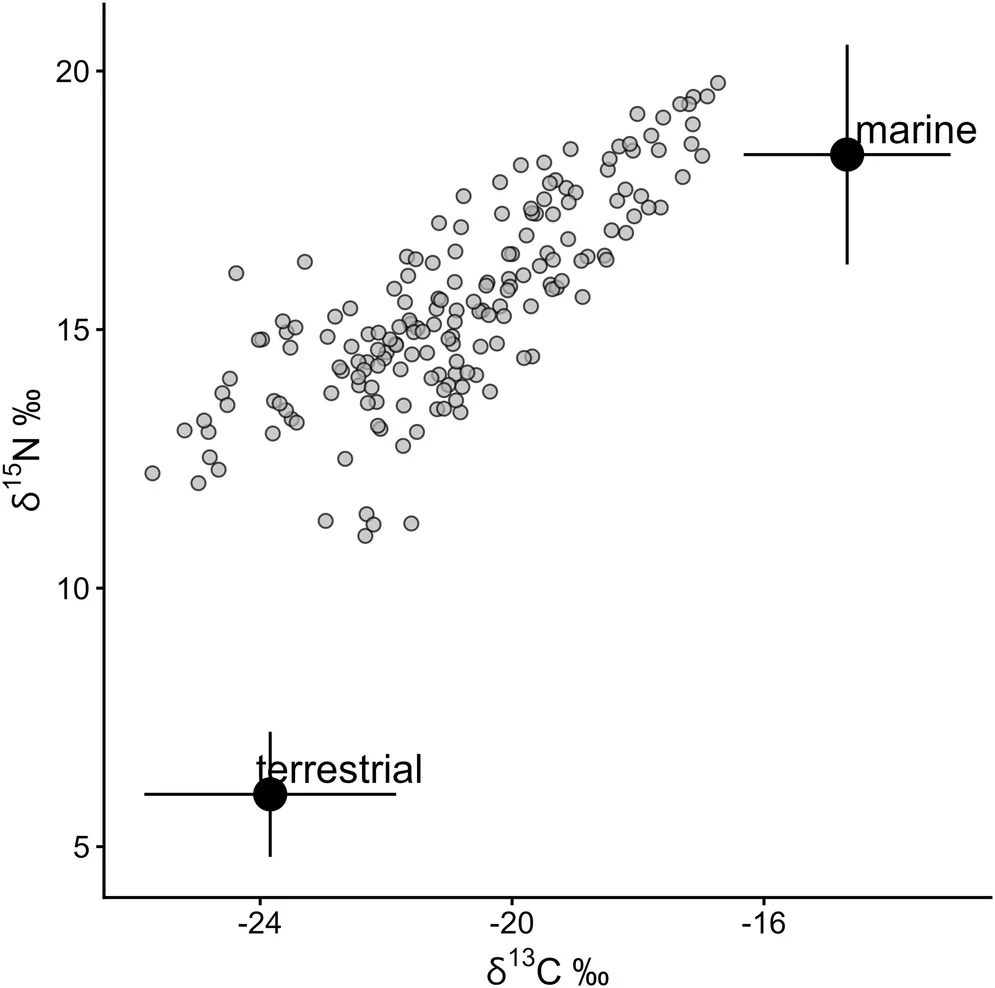
Isospace plot of δ^13^C and δ^15^N values of Herring gull chick downy feathers (points) relative to mean isotopic values of potential food sources. Source means (± SD) are corrected using trophic enrichment factors to allow comparison with consumer tissue.

We estimated the proportional contribution of marine versus terrestrial food sources using a Bayesian mixing model implemented in the *MixSIAR* package (v3.1.12; Stock et al. 2018) in R (v4.4.3; R Core Team 2025). Isotope values from food sources and trophic enrichment factors were primarily obtained from Sotillo et al. (2019), who determined the main marine and terrestrial food items for Lesser black‐backed gulls in the same ground colony. Because the diet of Herring gulls partially differs from that of Lesser black‐backed gulls (Kubetzki and Garthe 2003; Kim and Monaghan 2006), we refined the list of candidate food sources using long‐term regurgitation data collected from adults and chicks in the study colony. Based on these data, certain food sources from Sotillo et al. (2019) were excluded due to low relevance, and two additional anthropogenic food sources (pasta and ham) were incorporated. These items were freeze‐dried, ground and analysed for stable isotope ratios alongside feather samples. A full list of included food sources is provided in Table A2. Models were run with three Markov‐Chain Monte‐Carlo (MCMC) chains of 1,000,000 iterations. Model convergence was assessed using the Gelman‐Rubin (Gelman and Rubin 1992) and Geweke (Geweke 1992) diagnostics. The isospace plot is presented in Figure A1.

### 2.6 Statistical Analyses

We tested whether rooftop‐ and ground‐breeding pairs differed in local nest density and maternal quality, as these factors could potentially explain variation in yolk testosterone allocation. All analyses were performed in R v4.4.3. We fitted separate mixed‐effects models, all including breeding context (ground‐breeding or rooftop‐breeding), year and their interaction as fixed effects, and Pair ID as a random intercept, using the *lme4* package (v1.1–36; Bates et al. 2015) and the *glmmTMB* package (v1.1.10, Brooks et al. 2017). Nest density was analysed using a generalised linear mixed‐effects model with a Poisson error distribution and log link function. Maternal quality was evaluated using three complementary proxies: mean egg volume per nest, laying date of the last egg (day of the year) and the proportion of marine food sources in the diet. Egg volume and laying date were analysed using linear mixed‐effects models; diet proportion data were analysed using a beta‐distributed generalised linear mixed‐effects model with a logit link function. When diet data from multiple chicks per nest were available (55 of 79 cases), we used the mean proportion of marine resources per nest, justified by high within‐nest similarity in diet (ICC = 0.90). Diet data were unavailable in four cases. Significance of fixed effects was assessed using Type III ANOVA (*car* package v3.1–3; Fox and Weisberg 2019). Model assumptions, including overdispersion for the Poisson model, were evaluated using simulated residual diagnostics from the *DHARMa* package (v0.4.7; Hartig 2024). All assumptions were met.

To test whether yolk testosterone concentrations differ between rooftop‐ and ground‐breeding pairs, we fitted a linear mixed‐effects model with testosterone concentration (pg/mg) as the response variable, and breeding context, year and their interaction as fixed effects. Pair ID was included as a random intercept to account for repeated sampling of the same pairs across years. Significance of fixed effects was assessed using Type III ANOVA with Satterthwaite approximations. Because the breeding context × year interaction was significant, we performed post hoc pairwise comparisons using estimated marginal means with Tukey adjustment for multiple testing (*emmeans* package v1.11.0; Lenth 2025). Model assumptions were evaluated by visual inspection of simulated residuals with *DHARMa* and were met.

Next, we directly assessed the influence of local nest density and maternal quality on yolk testosterone allocation, by fitting a linear mixed‐effects model. Continuous predictors were standardised. Fixed effects included the number of neighbouring nests within 5 m, proportion of marine food sources, egg volume, laying date and year. Pair ID was included as a random intercept. Collinearity among predictors was assessed using variance inflation factors, which were all < 2, indicating low collinearity among predictors. Model assumptions were evaluated using simulated residual diagnostics with the *DHARMa* package, and all assumptions were met.

Lastly, because testosterone concentrations differed between years for the ground‐breeding colony, we conducted additional analyses restricted to ground‐breeding pairs sampled in both years (*n* = 7). To test whether the observed difference between years in the ground colony was also reflected in intra‐individual changes, we used a paired *t*‐test comparing testosterone concentrations between 2023 and 2024 within individuals. Normality of the difference scores (2024–2023) was assessed using visual inspection of histograms and Q‐Q plots and a Shapiro–Wilk test; assumptions were met. To examine whether within‐individual changes in yolk testosterone were associated with changes in nest density, diet, mean egg volume and laying date, we calculated difference scores for each variable (2024–2023) per pair and assessed associations using Spearman rank correlations, which are robust to small sample sizes and do not assume normally distributed data.

## 3 Results

**FIGURE 1:**
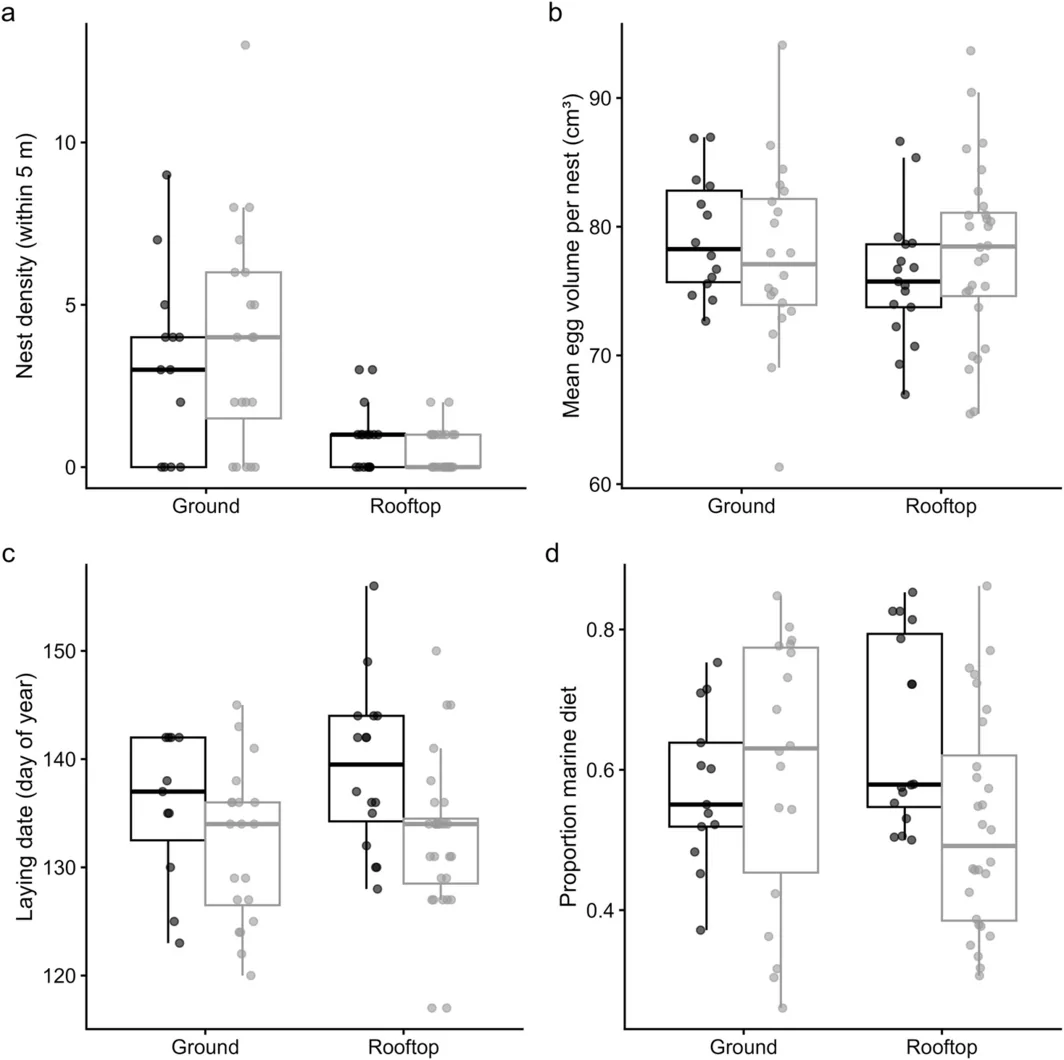
Differences in social environment and maternal quality proxies between ground‐ and rooftop‐breeding Herring gulls. Points are coloured by year (dark grey = 2023; light grey = 2024). (a) Nest density, expressed as the number of neighbouring nests within a 5 m radius. (b) Mean egg volume per nest. (c) Laying date (day of year). (d) Proportion of marine‐derived food sources in the diet. Rooftop‐breeding pairs experienced lower nest densities than ground‐breeding pairs, whereas no significant differences between breeding contexts were observed for maternal quality proxies.

Rooftop‐breeding pairs experienced significantly lower nest densities than ground‐breeding pairs (*χ* ^2^ = 10.67, *p* = 0.001), with no difference between years (*χ* ^2^ = 0.02, *p* = 0.90) and no breeding context × year interaction (*χ* ^2^ = 1.53, *p* = 0.22; *n* = 78 observations from 67 unique pairs) (Figure 1a). On average, rooftop pairs had 0.62 ± 0.81 neighbours within a 5 m radius, compared to 3.61 ± 3.20 neighbours for ground‐breeding pairs. Mean egg volume and laying date did not differ significantly between breeding contexts (egg volume: *χ* ^2^ = 0.38, *p* = 0.54; laying date: *χ* ^2^ = 0.30, *p* = 0.58) or years (egg volume: *χ* ^2^ = 0.002, *p* = 0.97; laying date: *χ* ^2^ = 2.33, *p* = 0.13), and no breeding context × year interactions were detected for either variable (egg volume: *χ* ^2^ = 0.03, *p* = 0.87; laying date: *χ* ^2^ = 0.12, *p* = 0.73) (Figure 1b,c; egg volume: *n* = 79 observations from 68 unique pairs; laying date: *n* = 75 observations from 64 unique pairs). The proportion of marine‐derived food sources in the diet ranged from 26% to 86% (mean ± SD: 58% ± 16%), indicating substantial variation among breeding pairs. However, female diet composition did not differ between breeding contexts (*χ* ^2^ = 1.14, *p* = 0.29) or years (*χ* ^2^ = 0.11, *p* = 0.74) (Figure 1d). The breeding context × year interaction was not significant (*χ* ^2^ = 3.59, *p* = 0.06; *n* = 75 observations from 65 unique pairs), but there was a tendency for rooftop breeding pairs to have a lower proportion of marine resources in 2024 compared to 2023.

**FIGURE 2:**
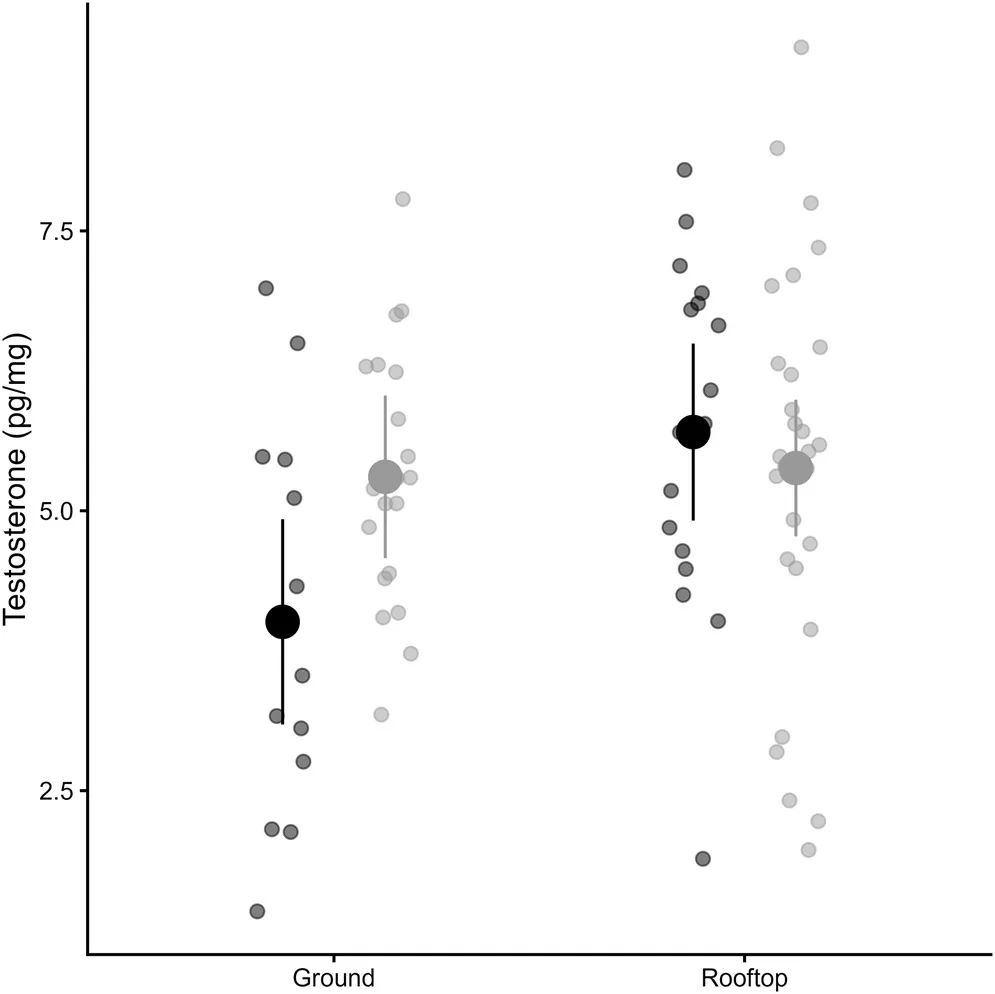
Yolk testosterone concentrations in eggs laid in a ground‐breeding colony and on urban rooftops across years. Points are coloured by year (dark grey = 2023; light grey = 2024). Smaller points represent raw data, while larger points with error bars represent model‐estimated marginal means ± 95% confidence intervals. Eggs from clutches laid on rooftops had significantly higher testosterone concentrations in 2023. Eggs in the ground colony differed significantly in yolk testosterone concentration across years, with higher concentrations in 2024 than in 2023, while concentrations remained similar across years for eggs laid by females breeding on rooftops.

There was a significant breeding context × year interaction effect on yolk testosterone concentration (*χ* ^2^ = 4.61, *p* = 0.03; *n* = 78 observations from 67 unique pairs; Figure 2). Eggs laid by rooftop‐ and ground‐breeding pairs differed in testosterone concentration in 2023, when eggs on rooftops contained higher testosterone levels than eggs from the ground colony (2023: ground mean ± SE = 4.01 ± 0.46 pg/mg, roof mean ± SE = 5.70 ± 0.40 pg/mg, *p* = 0.007; 2024: ground mean ± SE = 5.30 ± 0.36, roof mean ± SE = 5.38 ± 0.31, *p* = 0.87). Eggs from the ground colony also differed significantly in testosterone concentrations between years (mean ± SE: 2023 = 4.01 ± 0.46 pg/mg, 2024 = 5.30 ± 0.36 pg/mg; *p* = 0.03), whereas yolk testosterone concentrations of eggs laid on rooftops remained similar across years (mean ± SE: 2023 = 5.70 ± 0.40 pg/mg, 2024 = 5.38 ± 0.31 pg/mg; *p* = 0.52).

**FIGURE A2:**
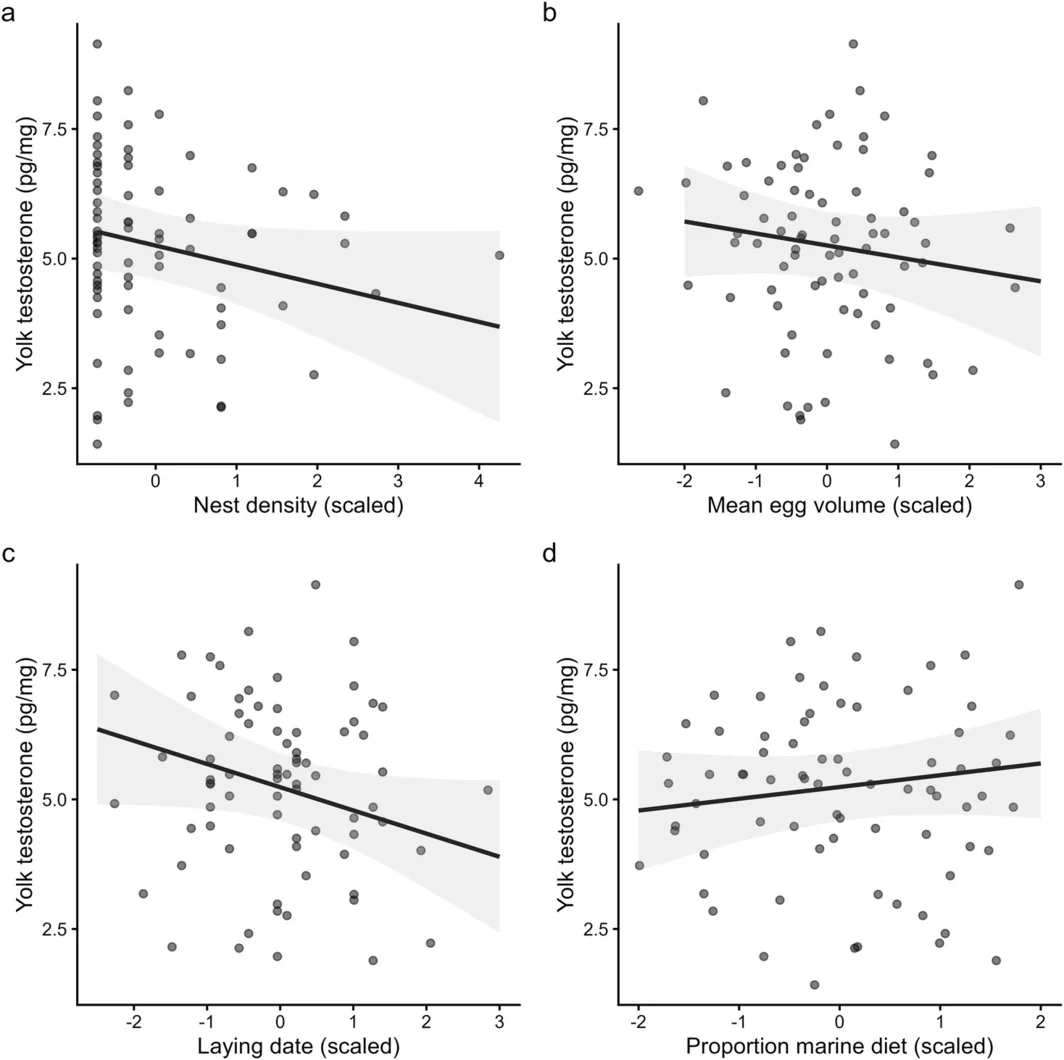
Partial effects of (a) nest density, (b) mean egg volume, (c) laying date and (d) proportion of marine diet on yolk testosterone concentrations. Lines show predictions from the linear mixed‐effects model while holding all other predictors constant at their mean values; shaded areas indicate 95% confidence intervals. Points represent raw observations.

Yolk testosterone concentrations were not significantly affected by year, diet or egg volume. Nest density and laying date were not significantly associated with yolk testosterone concentration, but there was a tendency towards lower testosterone concentrations with increasing nest density and later laying dates (Table 1; Figure A2).

**TABLE 1:** Linear mixed‐effects model results of the effect of social environment and maternal quality proxies on yolk testosterone concentration.

	Estimate ± SE	*χ* ^2^	*p*
Nest density (scaled)	−0.37 ± 0.21	3.17	0.08
Proportion marine diet (scaled)	0.23 ± 0.22	1.02	0.31
Year (2024; ref. = 2023)	−0.09 ± 0.38	0.05	0.82
Mean egg volume (scaled)	−0.23 ± 0.21	1.15	0.28
Laying date (scaled)	−0.45 ± 0.24	3.51	0.06

*Note:* *N* = 71 observations from 61 unique pairs.

**FIGURE A3:**
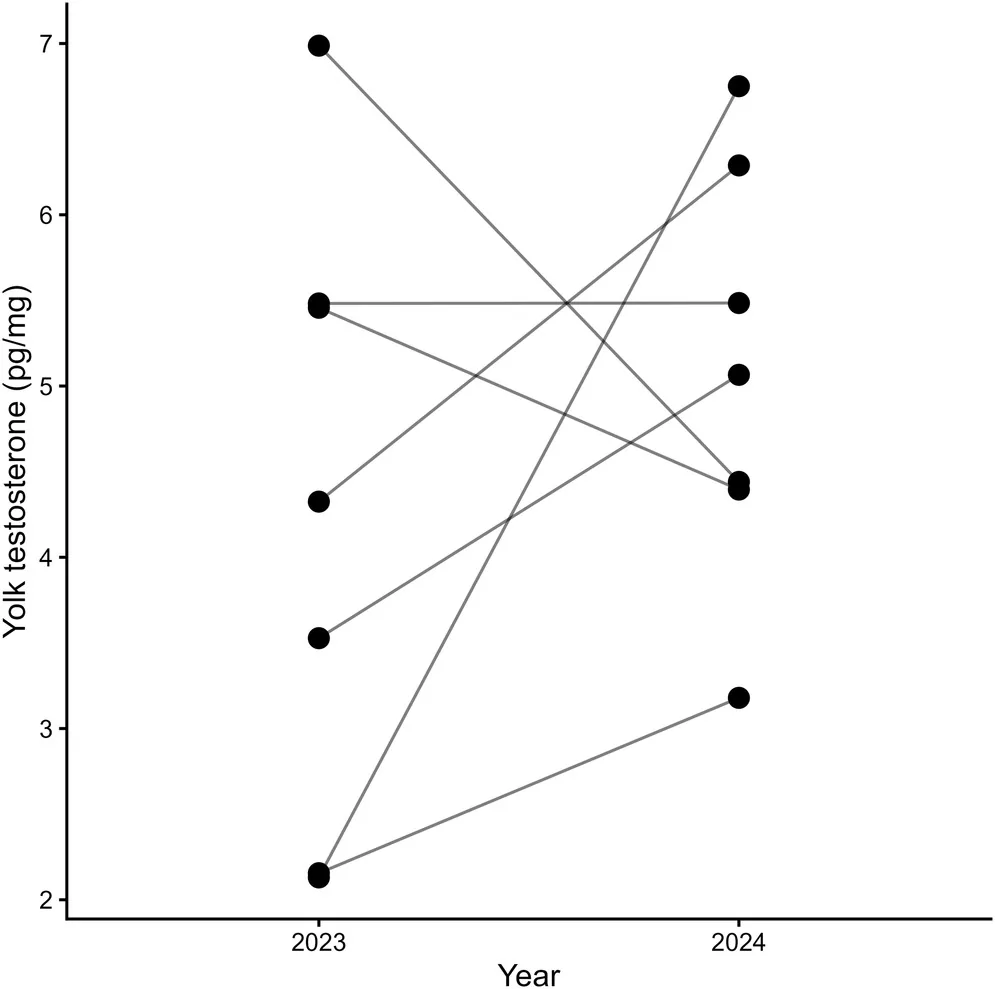
Within‐female variation in yolk testosterone concentrations between 2023 and 2024 in ground‐breeding Herring gulls. Lines connect measurements from eggs of the same pairs sampled in both years (*n* = 7).

Because yolk testosterone concentrations varied between years in the ground colony, we further examined whether this population‐level pattern was reflected in intra‐individual changes between years and whether within‐pair changes in environmental or intrinsic factors were associated with changes in testosterone levels across years. For pairs sampled in both years in the ground colony (*n* = 7), yolk testosterone concentrations showed no consistent within‐female change between years (*t*_6_ = −0.91, *p* = 0.40; Figure A3). The mean within‐female change (2024–2023) was −0.79 ± 2.30 pg/mg (95% CI: −2.92–1.34). Changes in yolk testosterone concentrations over years were also not significantly correlated with changes in nest density (Spearman's *ρ* = −0.22, *p* = 0.63), egg volume (*ρ* = −0.43, *p* = 0.35), laying date (*ρ* = −0.20, *p* = 0.67) or diet composition (*ρ*  = −0.31, *p* = 0.56; *n* = 6).

## 4 Discussion

In this study, we investigated whether maternal allocation of yolk testosterone differs between rooftop‐ and ground‐breeding Herring gulls and aimed to identify the factors underlying such variation. Yolk testosterone allocation differed between rooftop‐ and ground‐breeding pairs in 2023, but contrary to our predictions, eggs from rooftop‐breeding pairs contained higher testosterone levels despite lower nest densities. In fact, we found no relationship between yolk testosterone levels and nest density. Additionally, we found no differences between breeding contexts in our proxies of maternal condition (egg volume, laying date and diet), nor were these associated with yolk testosterone allocation. Variation in yolk testosterone allocation likely reflects processes that were not explicitly investigated in this study, given that rooftop‐ and ground‐breeding contexts differ in multiple ecological characteristics, with possibly greater interannual environmental variability in ground colonies.

### 4.1 Rooftops as Novel Social Environments

Previous studies have identified social stimuli, often quantified as breeding density, as strong ecological predictors of yolk testosterone levels, with increased social interactions typically associated with elevated testosterone (reviewed in James and Bentz 2024). However, this expected relationship was not supported in our study. Despite differences in social context between rooftop‐ and ground‐breeding pairs, nest density did not explain variation in yolk testosterone allocation. This suggests that, in this population, nest density is unlikely to be a primary determinant of testosterone deposition.

In colonies, where offspring are expected to experience frequent social interactions, the potential benefits of elevating offspring aggression via increased testosterone may be reduced, as further increases in testosterone could heighten the risk of injury or social conflict, potentially reducing reproductive success. Indeed, a meta‐analysis by Bentz et al. (2016) showed that the effect of competition on yolk testosterone is generally weaker or absent in colonial species compared to solitary or less aggregated species. Intriguingly, in a study on Lesser Black‐backed gulls in the same ground‐breeding colony, Salas et al. (2022) found that eggs laid in high‐density parts of the colony produced less explorative chicks compared to eggs laid in low‐density parts, showing that mothers adjust offspring phenotype to high‐density colonial environments, likely to reduce agonistic interactions with conspecifics. However, the underlying mechanisms, including a potential role for yolk testosterone, were not assessed.

Alternatively, direct social interactions may be more relevant for explaining variation in maternal hormone deposition. Physical proximity does not necessarily reflect the frequency or intensity of competitive interactions, particularly in colonial contexts, where cooperative or altruistic behaviour towards neighbours can enhance fitness (Lewis et al. 2007). Supporting this idea, García et al. (2025) reported lower testosterone concentrations in eggs from blue tit pairs exposed to playbacks simulating higher perceived social density without increased social interactions compared to control pairs, suggesting that females may adjust offspring phenotype to become more sociable rather than aggressive when social cues do not reflect heightened competition. Together, these findings suggest that although yolk testosterone is not associated with nest density per se in our study population, this does not rule out a role for social environment, but rather indicates that density may not fully capture the relevant social interactions influencing maternal hormone allocation. Future work should therefore incorporate direct behavioural observations of aggression or competitive interactions, rather than relying solely on nest density.

### 4.2 Female Quality and Testosterone Allocation

Additionally, we predicted that females in poorer condition may compensate for developmental constraints of their offspring by depositing higher levels of yolk testosterone (Verboven et al. 2003). While we expected that rooftop colonies may disproportionally attract competitively inferior individuals unable to secure nesting sites in dense ground colonies, none of the proxies for female quality that we measured differed between rooftop‐ and ground‐breeding pairs, suggesting that the aspects of maternal quality captured by these proxies were largely uniform across breeding contexts.

One possible explanation is that, although rooftops may initially have been primarily used by lower‐quality individuals, this effect may diminish over time as populations become established. In Belgium, Ostend was the first city where Herring gulls were observed breeding on rooftops, with the first indications of rooftop breeding in 1993 and the first confirmed breeding cases recorded in 1998 (François 2002). The rooftop‐breeding population subsequently increased from 33 breeding pairs in 1998 to 205 pairs in 2001 (François 2002; Stienen et al. 2002), and continued to increase during the following years (Stienen and Courtens 2010). By 2025, 768 Herring gull nests were recorded on rooftops in Ostend and its immediate surroundings (INBO, unpublished data). Given the high site fidelity in this species, with individuals typically returning to the same breeding location each year, any initial differences in breeder quality between rooftop and ground habitats may have eroded over time. Additionally, the ongoing loss of natural habitats may increasingly force even high‐quality individuals to breed on rooftops. Alternatively, rooftops may constitute a viable alternative breeding strategy, not only selected by competitively inferior individuals. Rooftop nesting sites may differ from traditional ground colonies in the costs and benefits they entail. The rooftops in Ostend combine a close proximity to marine foraging areas with access to anthropogenic food sources and reduced predation risk from ground predators, while they may impose higher costs for incubation and reduce hatching success (Van Malderen et al. 2026). As a result, these contrasting costs and benefits may not lead to strong habitat‐dependent differences in female quality.

Furthermore, none of the measured proxies for female quality were related to yolk testosterone concentrations, offering no support for a compensatory response by females in poorer condition. This contrasts with previous findings in Lesser black‐backed gulls, where females in poorer body condition laid eggs with higher testosterone levels (Verboven et al. 2003). However, this study relied on body weight relative to size and the size of pectoral muscles to determine maternal condition. Because we did not measure females directly, we relied on different proxies of maternal condition that could not be corrected for female body size. Although higher‐quality females generally produce larger eggs and lay earlier (Murphy 1986; Reid and Boersma 1990; Moreno et al. 1998; Descamps et al. 2011), and marine food sources are considered of higher nutritional value for Herring gulls (Pierotti and Annett 1991), these proxies may not capture all aspects of maternal condition relevant to hormone deposition. Incorporating direct measures of body size or condition could help clarify the relationship between intrinsic state and yolk testosterone deposition. In this context, it would also be valuable to assess the within‐clutch variation in yolk testosterone levels, which is thought to modulate brood reduction (Eising et al. 2001; Müller et al. 2004), and may hence vary with female quality and environmental conditions. Although using a single egg from the same laying order provides a standardised comparison among breeding pairs, it does not allow us to determine whether rooftop‐ and ground‐breeding females differ in how testosterone is allocated across the laying sequence.

### 4.3 Temporal Variation in Yolk Testosterone Allocation

Because differences in yolk testosterone concentration between breeding contexts were not consistent with variation in nest density or maternal quality, and were not consistent across years, our results suggest that additional, unmeasured intrinsic or extrinsic factors may play a more prominent role in determining yolk testosterone allocation. Both the difference between rooftop and ground habitats observed in 2023 and the interannual differences among ground‐breeding pairs seem to be driven by comparatively low testosterone concentrations in ground‐breeding pairs in 2023, whereas levels in 2024 were similar to those measured on rooftops. This pattern indicates that temporal variation in environmental factors within the ground colony, rather than consistent differences between rooftop‐ and ground‐breeding contexts, may underlie the observed breeding context × year interaction. This may reflect differences in environmental variability between breeding contexts, with ground colonies potentially experiencing greater interannual fluctuations, whereas rooftop environments may be comparatively stable environments. For example, vegetation structure in the ground colony may vary between years due to weather conditions, with increased vegetation growth in wetter years potentially obstructing visual contact among neighbours and thereby reducing perceived local density. In contrast, unvegetated rooftop environments may remain largely free of such variation. Because visibility influences the type and frequency of aggressive interactions in breeding gulls (Burger 1977; Bukacinska and Bukacinski 1993), such habitat‐specific differences in visual obstruction may ultimately influence yolk testosterone allocation. Future studies including multiple ground‐breeding colonies across multiple years could help determine whether the interannual variation observed here reflects a broader pattern of ground‐breeding environments or is specific to this colony.

Importantly, repeated sampling of ground‐breeding pairs revealed no consistent directional shift in yolk testosterone concentrations within females across years, indicating that the population‐level interannual pattern was not driven by consistent within‐individual shifts. Likewise, within‐female changes in yolk testosterone concentration were not significantly correlated with changes in nest density, diet composition, egg volume or laying date, suggesting that the interannual shift cannot be attributed to changes in these measured variables. Instead, the results point towards broader colony‐level or environmental processes that varied between years, although the small number of repeated pairs (*n* = 7) substantially limits statistical power, so that biologically meaningful within‐female changes or associations cannot be excluded. These results should therefore be interpreted cautiously.

## 5 Conclusion

Our results demonstrate that yolk testosterone allocation in Herring gulls is not consistently associated with breeding context, nest density or maternal quality, but instead varies across years in a context‐dependent manner. This suggests that maternal hormone deposition in our study population is variable, yet not tightly linked to differences between rooftop‐ and ground‐breeding contexts or commonly used ecological proxies. Because rooftop‐ and ground‐breeding contexts differ in multiple ecological characteristics, identifying the specific environmental cues driving maternal testosterone allocation remains an important challenge. Alternatively, given that rooftops represent evolutionarily novel habitats for Herring gulls, maternal endocrine strategies shaped under traditional ground‐breeding conditions may not yet be optimally adjusted to rooftop‐breeding contexts. A key remaining question is whether the observed variation has functional consequences for offspring. Linking yolk testosterone levels to offspring performance and fitness across contrasting breeding contexts will be essential to determine whether yolk testosterone allocation is functionally matched to local conditions or reflects physiological or environmental constraints. Disentangling adaptive adjustment from physiological constraint or potential mismatches in cue perception will require longer‐term studies across multiple years and breeding contexts, incorporating experimental approaches such as cross‐fostering.

## Author Contributions

**Jolien Van Malderen:** conceptualization (equal), data curation (lead), formal analysis (lead), funding acquisition (equal), investigation (lead), methodology (lead), project administration (equal), software (lead), validation (lead), visualization (lead), writing – original draft (lead), writing – review and editing (equal). **Luc Lens:** conceptualization (equal), funding acquisition (equal), project administration (equal), resources (equal), supervision (supporting), writing – review and editing (equal). **Frederick Verbruggen:** conceptualization (equal), funding acquisition (equal), project administration (equal), resources (equal), supervision (supporting), writing – review and editing (equal). **Eric W. M. Stienen:** conceptualization (equal), investigation (supporting), project administration (equal), resources (equal), supervision (supporting), writing – review and editing (equal). **Wendt Müller:** conceptualization (equal), formal analysis (supporting), methodology (supporting), project administration (equal), resources (equal), supervision (lead), writing – review and editing (equal).

## Funding

This work was supported by Universiteit Gent (01M00221). Bijzonder Onderzoeksfonds UGent (BOF22/DOC/019).

## Conflicts of Interest

The authors declare no conflicts of interest.

## Data Availability

Data and code supporting this study are available on Zenodo: doi:10.5281/zenodo.19677789. These will be published and made publicly available upon acceptance.

## Appendix A

**TABLE A1:** Overview of sampling locations and collected samples.

Sampling location	Breeding context	Latitude (°N)	Longitude (°E)	Year(s) monitored	Nr of eggs collected	Nr of eggs included in analysis
Zeebrugge ground colony	Ground	51.3458	3.1728	2023 + 2024	48	34
R1	Rooftop	51.2181	2.9039	2023	1	0
R2	Rooftop	51.2286	2.9183	2023	1	1
R3	Rooftop	51.2158	2.9127	2023	2	2
R4	Rooftop	51.2297	2.9257	2023	10	5
R5	Rooftop	51.2300	2.9260	2023 + 2024	16	9
R6	Rooftop	51.2325	2.9362	2023 + 2024	5	5
R7	Rooftop	51.2336	2.9177	2023 + 2024	5	4
R8	Rooftop	51.2325	2.9390	2024	10	7
R9	Rooftop	51.2330	2.93096	2024	21	11
R10	Rooftop	51.2327	2.9398	2024	1	1

*Note:* Eggs were excluded from analyses either because visible embryonic development was observed (*n* = 17), or because a subset of available eggs from rooftop sites with relatively large sample sizes was excluded to reduce imbalance in sample sizes between breeding contexts (*n* = 24).

**TABLE A2:** Food sources included in the mixing model for diet assessment.

Food sample	Category
Earthworm	Terrestrial
Chicken	Terrestrial
Bread	Terrestrial
Fried potato	Terrestrial
Crisps	Terrestrial
Ham	Terrestrial
Pasta	Terrestrial
Starfish	Marine
*Mytilus edulis*	Marine
*Carcinus maenas*	Marine
Shrimp	Marine
*Liocarcinus holsatus*	Marine
*Liocarcinus vernalis*	Marine
Fish mixture	Marine
*Gadus morhua*	Marine
